# Supplementary material for: Host-specific bacterial modulation of airway gene expression and alternative splicing
Source: mSphere. 2025 Oct 30;10(11):e00577-25. doi: 10.1128/msphere.00577-25 (PMC12646005; doi:10.1128/msphere.00577-25)
Supplement: Supplemental Material — Supplemental figures and table captions. [file msphere.00577-25-s0001.docx]

**Supplementary Figures**

**
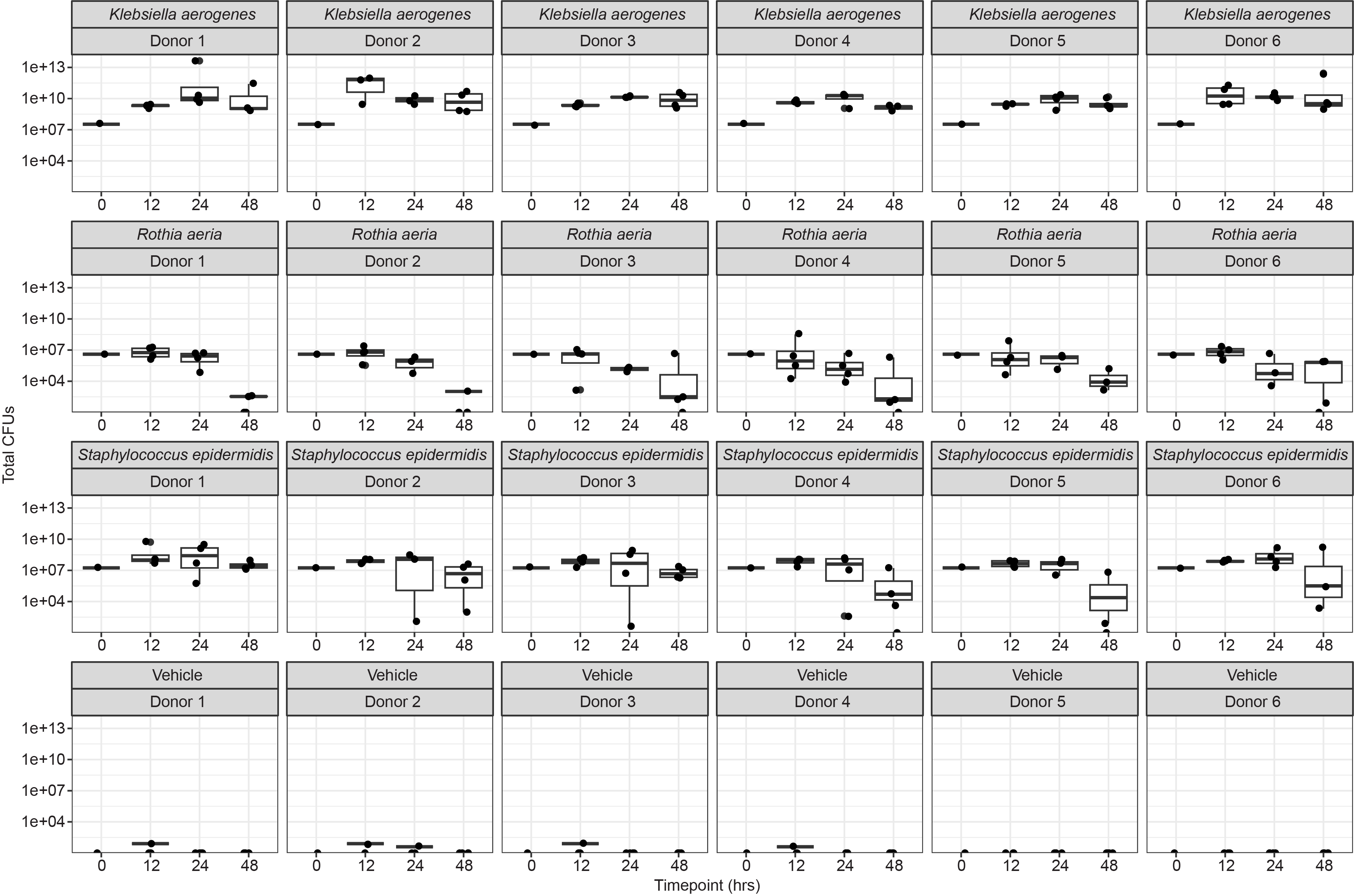
**

**Figure S1. Microbial CFUs of inoculum and at each endpoint.** At time of dosing (0 hours) and each time point (12 hours, 24 hours, and 48 hours), the bacterial inoculum or apical wash was serially diluted and plated on tryptic soy agar (TSA) to quantify colony forming units (CFUs). For boxplots, box middle represents the median, box edges represent 25^th^ and 75^th^ quartiles, and outlier values are separate points.

**
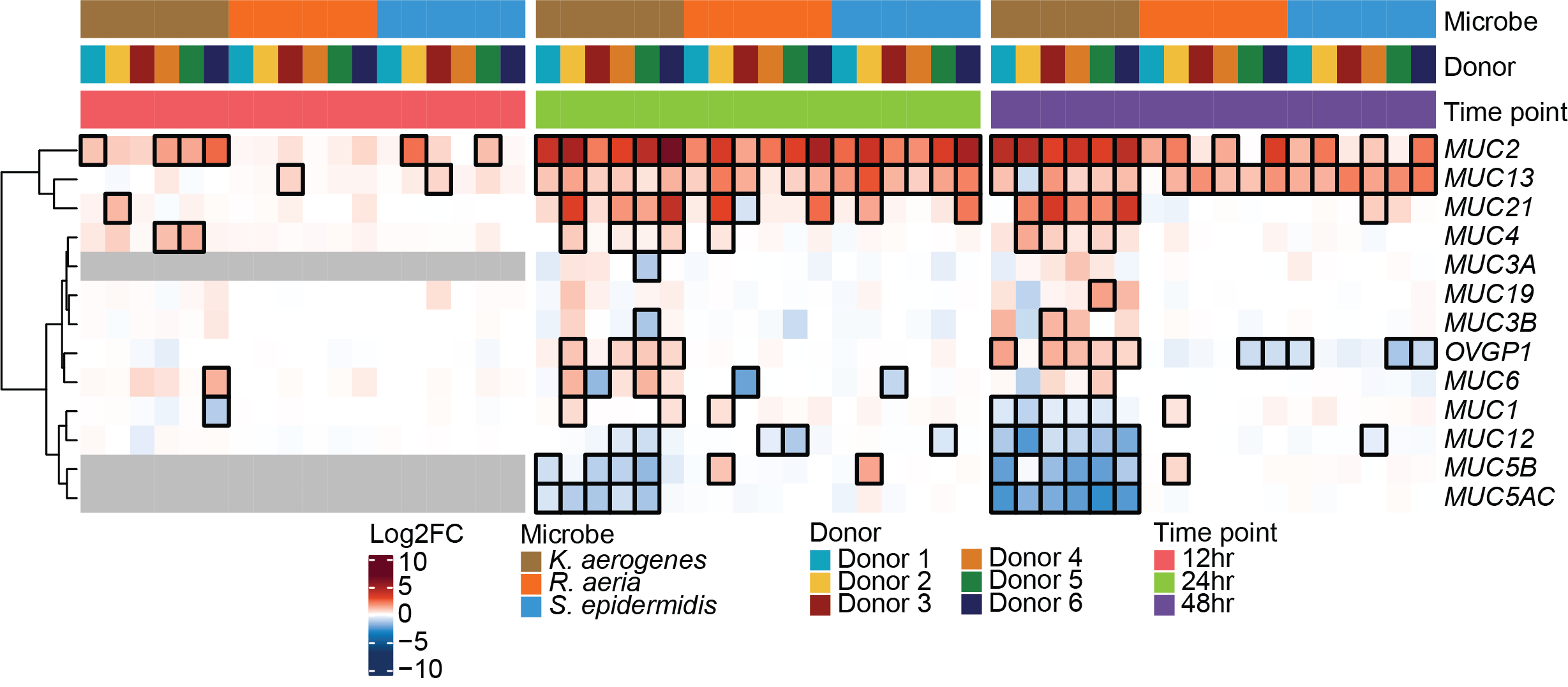
**

**Figure S2.** **Changes in gene expression of mucin-related genes.** Heatmap of mucin-related genes, which comprise mucus. Each row represents a different gene and each column a different sample. Columns were hierarchically clustered. Each column was annotated by color with the sample’s microbial treatment, donor cells, and time point. Cells were colored by the log_2_ fold change (log_2_FC) relative to the appropriate vehicle control. Outlined boxes have an adjusted P-value < 0.05. Gray boxes represent genes that were filtered out due to low gene counts at a specific time point. Genes that were not present in at least 2/3 time points were removed.

**
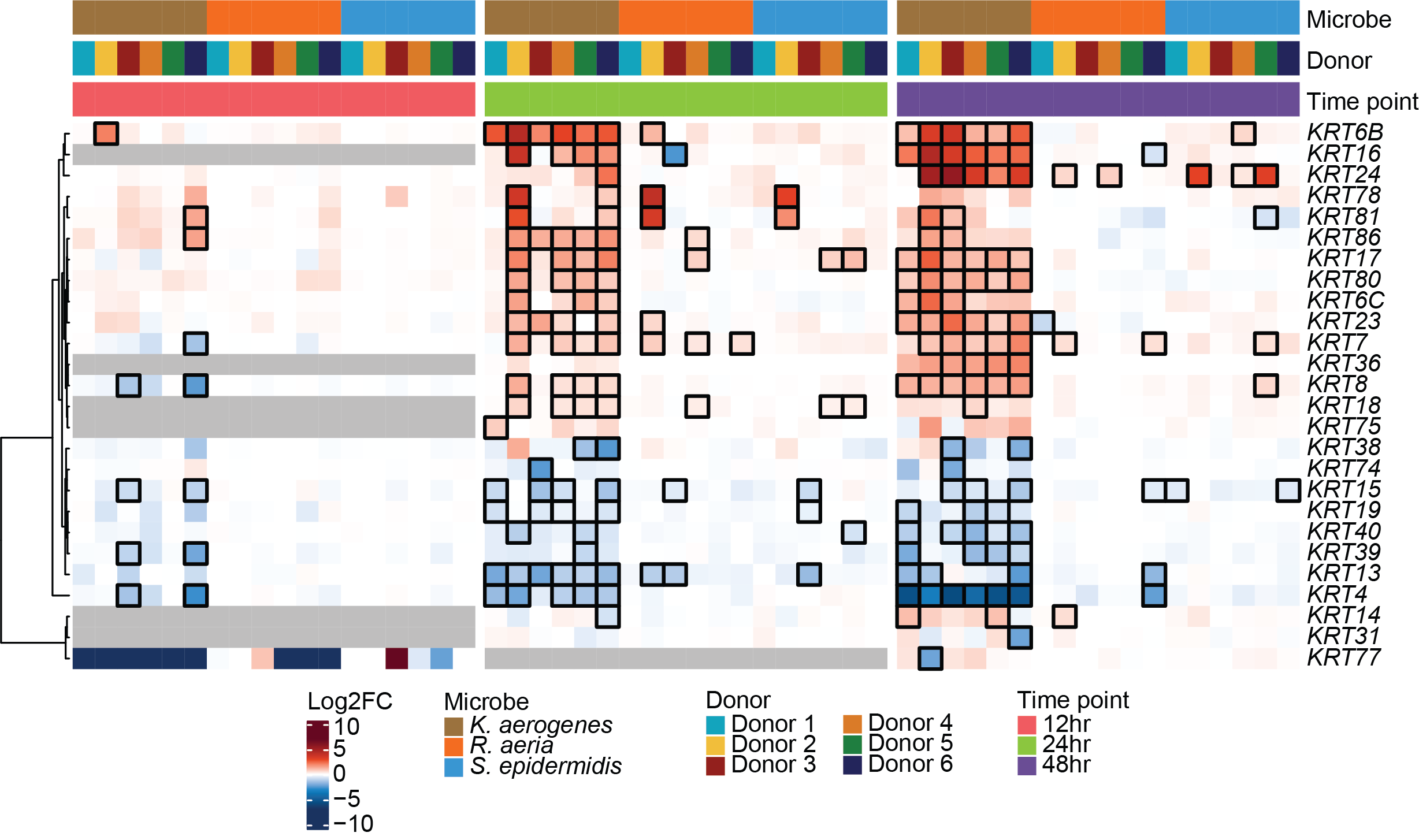
**

**Figure S3.** **Changes in gene expression of keratin genes.** Heatmap of keratin genes, which provide structural integrity. Each row represents a different gene and each column a different sample. Columns were hierarchically clustered. Each column was annotated by color with the sample’s microbial treatment, donor cells, and time point. Cells were colored by the log_2_ fold change (log_2_FC) relative to the appropriate vehicle control. Outlined boxes have an adjusted P-value < 0.05. Gray boxes represent genes that were filtered out due to low gene counts at a specific time point. Genes that were not present in at least 2/3 time points were removed.

**
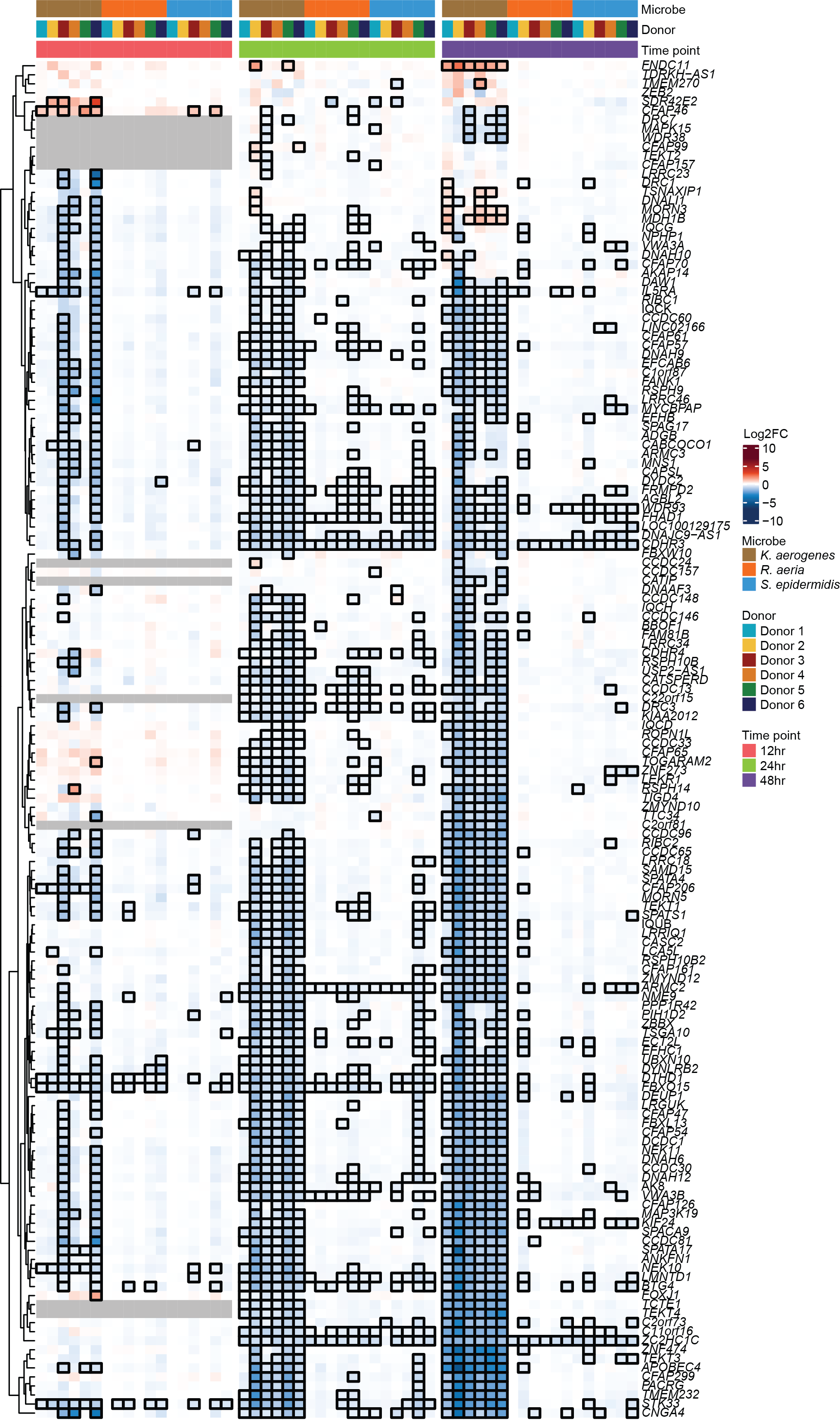
**

**Figure S4.** **Changes in gene expression of ciliary genes.** Heatmap of ciliary genes. Each row represents a different gene and each column a different sample. Columns were hierarchically clustered. Each column was annotated by color with the sample’s microbial treatment, donor cells, and time point. Cells were colored by the log_2_ fold change (log_2_FC) relative to the appropriate vehicle control. Outlined boxes have an adjusted P-value < 0.05. Gray boxes represent genes that were filtered out due to low gene counts at a specific time point. Genes that were not present in at least 2/3 time points were removed.

**
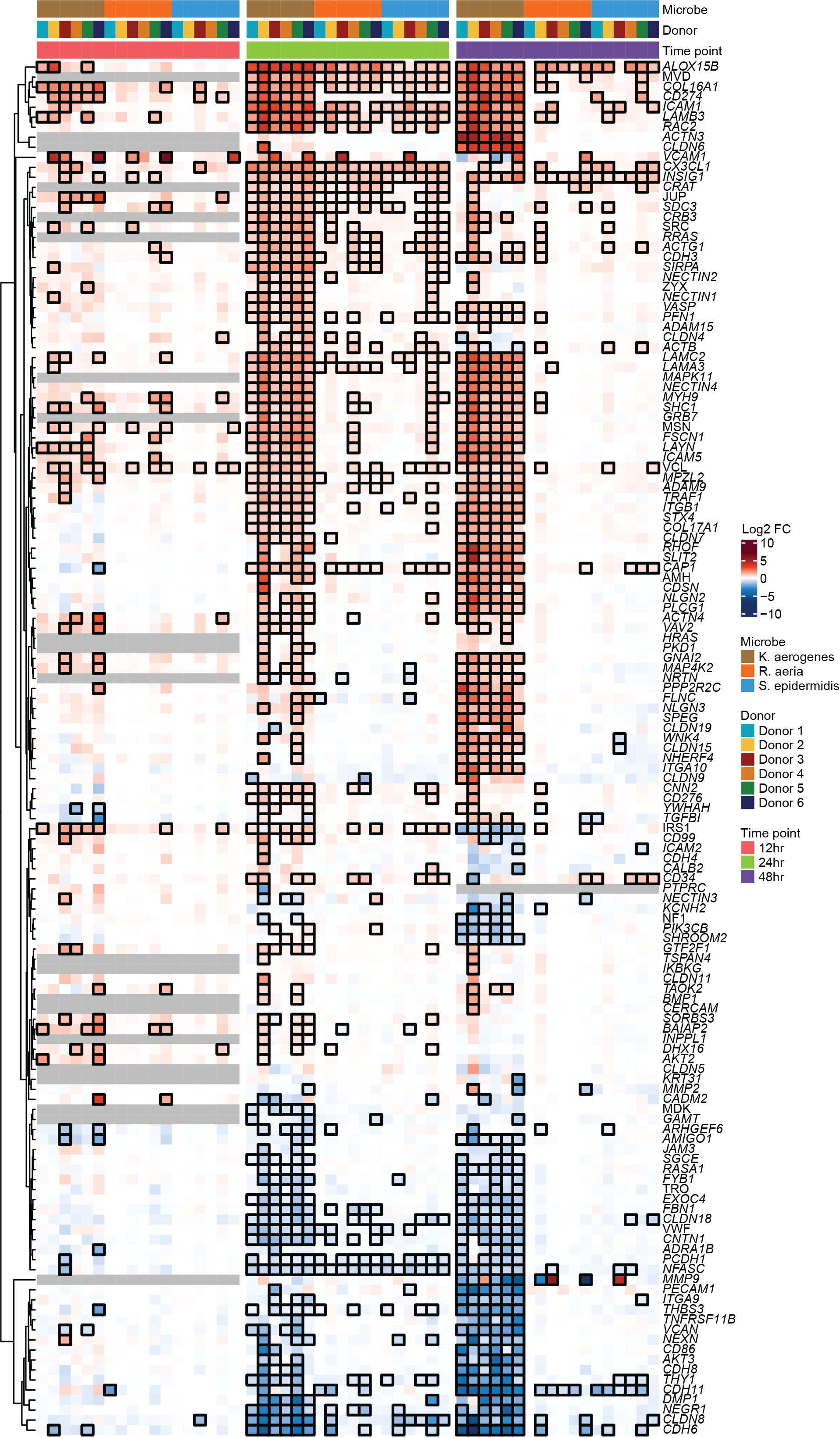
**

**Figure S5.** **Changes in gene expression of apical junction genes.** Heatmap of apical junction genes. Each row represents a different gene and each column a different sample. Columns were hierarchically clustered. Each column was annotated by color with the sample’s microbial treatment, donor cells, and time point. Cells were colored by the log_2_ fold change (log_2_FC) relative to the appropriate vehicle control. Outlined boxes have an adjusted P-value < 0.05. Gray boxes represent genes that were filtered out due to low gene counts at a specific time point. Genes that were not present in at least 2/3 time points were removed.

**
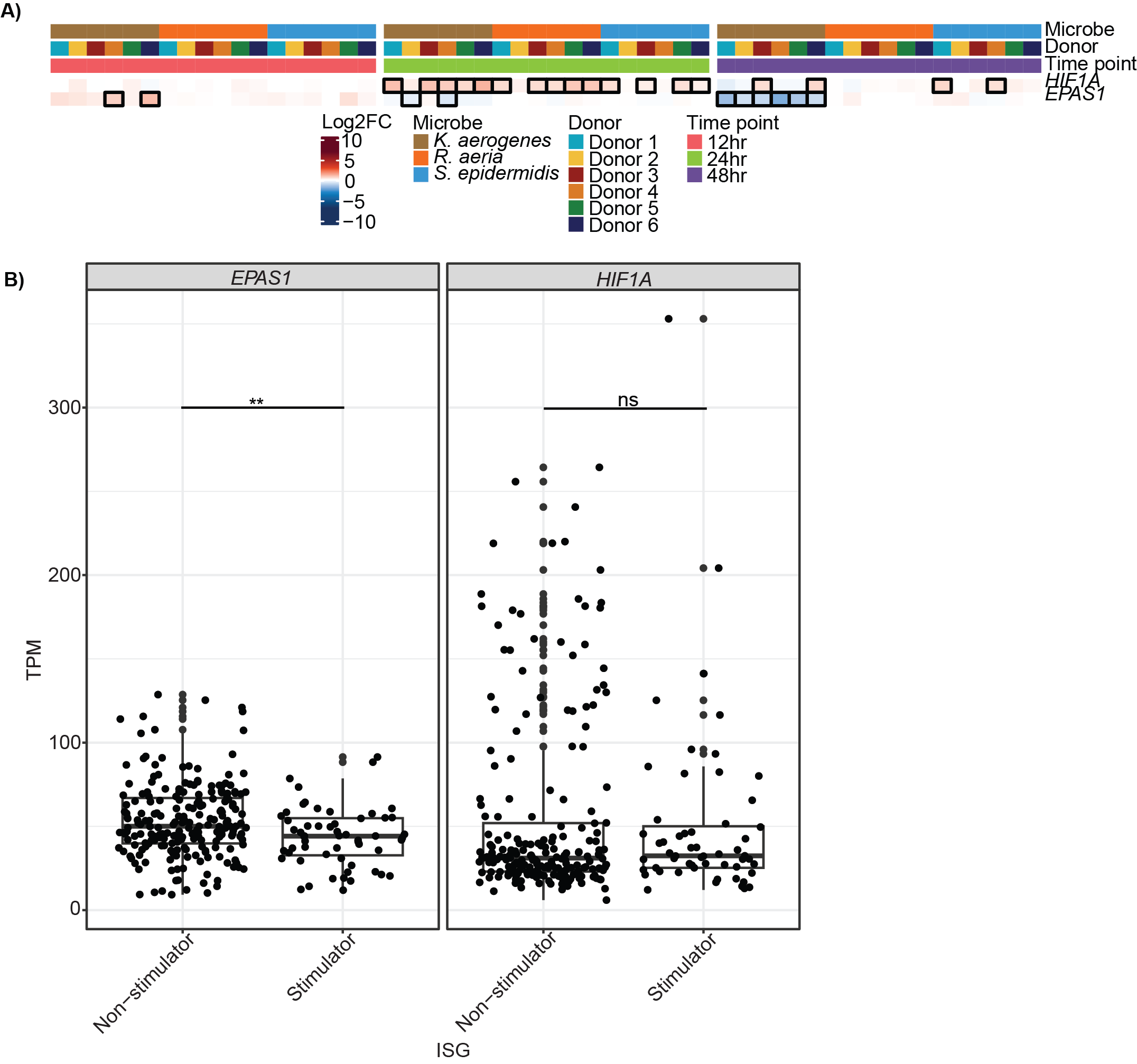
**

**Figure S6.** **Changes in gene expression of hypoxia genes. A)** Heatmap of *HIF-1α* and *EPAS1* (HIF-2α), the transcription factors activated by hypoxia. Each row depicts a different gene and each column a different sample. Samples are color annotated according to the donor, microbial treatment, and time point. Rows are hierarchically clustered. Cells are colored based on the log_2_ fold change. Outlined cells represent an FDR-adjusted P-value < 0.05. **B)** Boxplots of TPM (transcripts per million) for *HIF-1α* and *EPAS1*. Comparison using Wilcoxon test. For relevant plots, * represented P-value < 0.05, ** P-value < 0.01, *** P-value < 0.001, and ns = non-significant. For boxplots, box middle represents the median, box edges represent 25^th^ and 75^th^ quartiles, and outlier values are separate points.


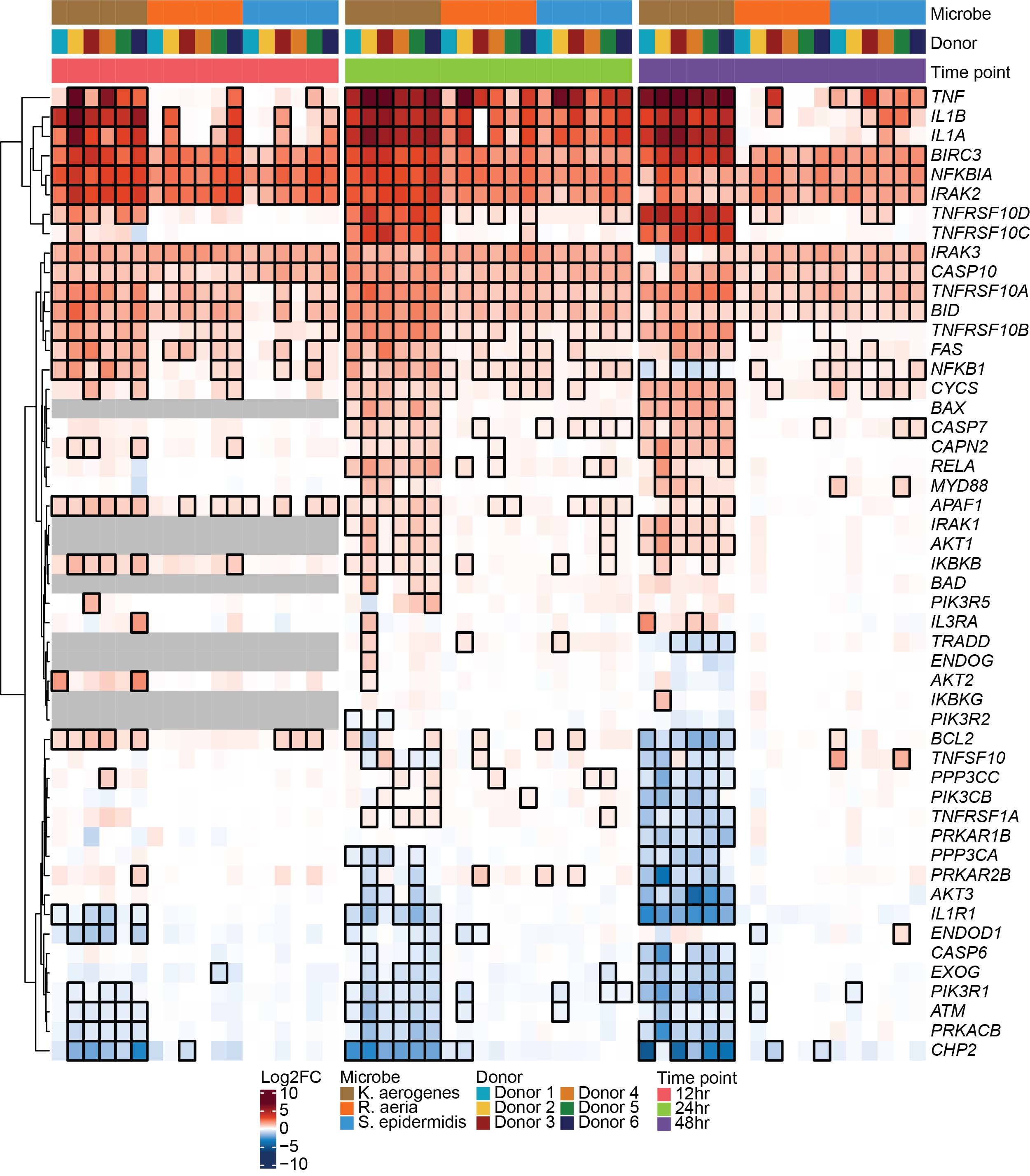


**Figure S7.** **Changes in gene expression of apoptosis-related genes.** Heatmap of apoptosis-related genes. Each row represents a different gene and each column a different sample. Columns were hierarchically clustered. Each column was annotated by color with the sample’s microbial treatment, donor cells, and time point. Cells were colored by the log_2_ fold change (log_2_FC) relative to the appropriate vehicle control. Outlined boxes have an adjusted P-value < 0.05. Gray boxes represent genes that were filtered out due to low gene counts at a specific time point. Genes that were not present in at least 2/3 time points were removed.


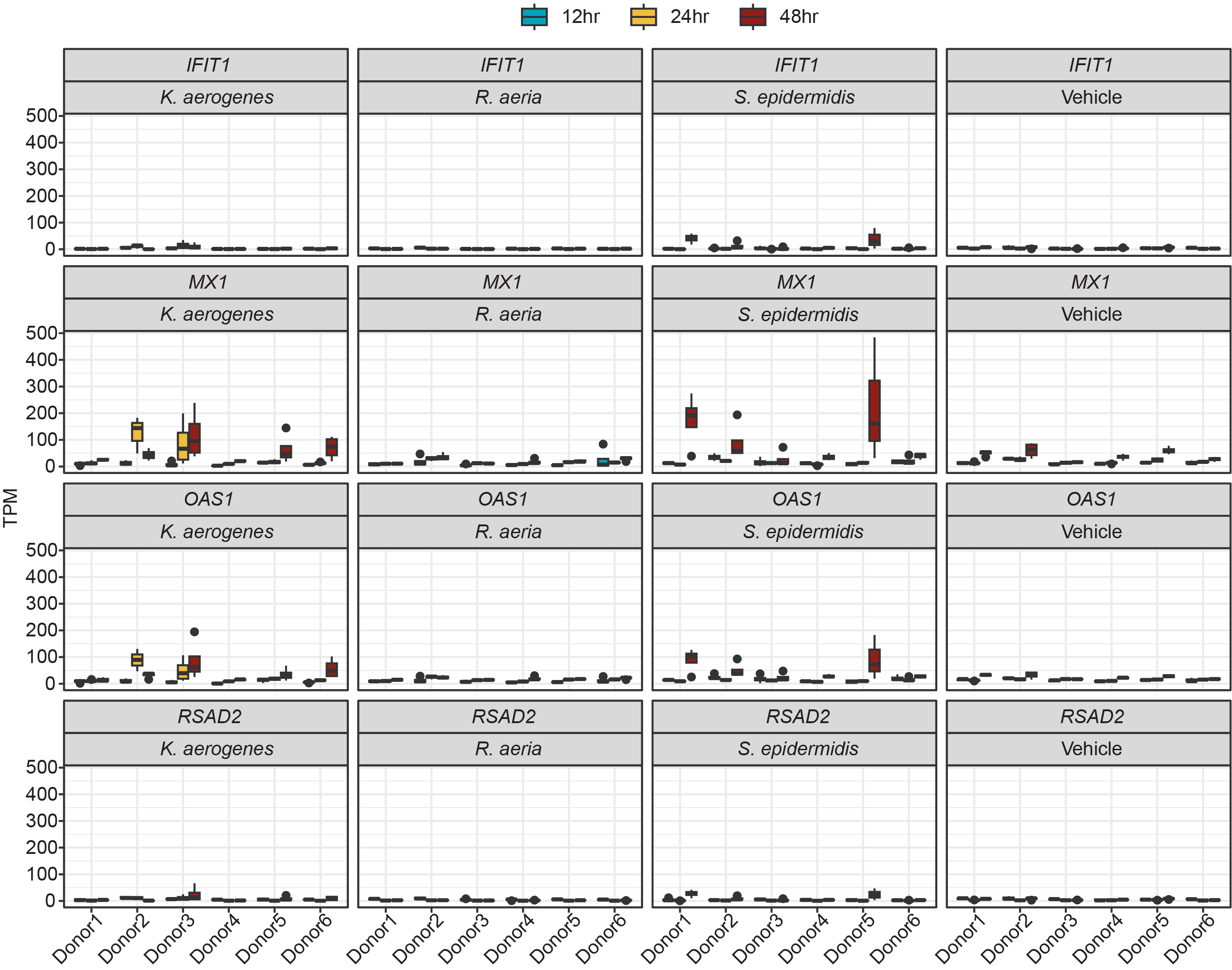


**Figure S8. Gene expression of ISGs following microbial treatment versus vehicle.** Boxplot of TPM (normalized gene counts) for select ISGs in each microbial treatment and vehicle control. Box middle represents the median, box edges represent 25^th^ and 75^th^ quartiles, and outlier values are separate points. For boxplots, box middle represents the median, box edges represent 25^th^ and 75^th^ quartiles, and outlier values are separate points.


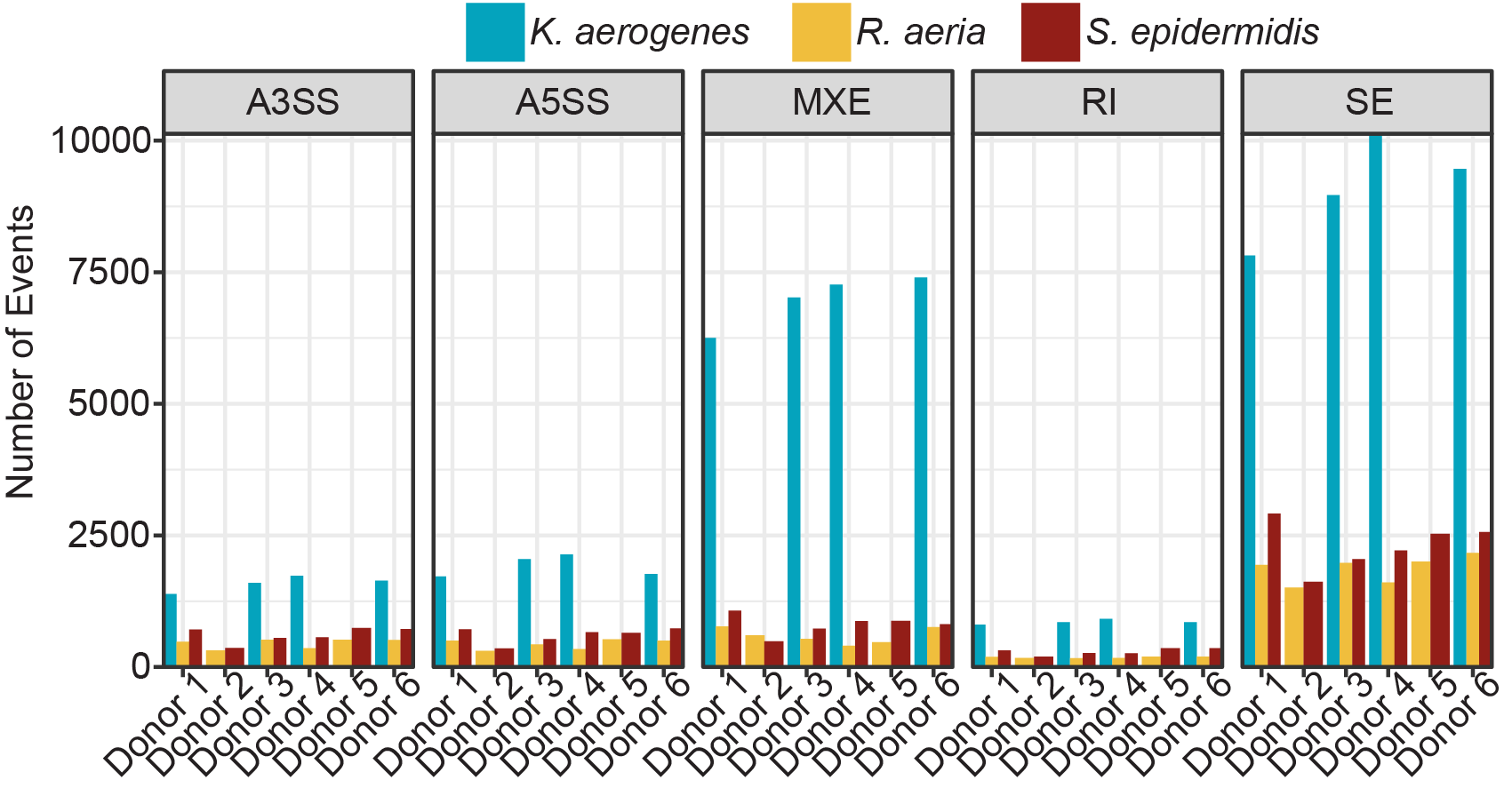


**Figure S9. Distribution of differential splicing events.** Plot of the number of statistically significant differential splicing events (|ΔPSI| > 0.1 and FDR < 0.05) for each microbe and donor, shown per event type (A3SS: alternative 3’ splice site, A5SS: alternative 5’ splice site, MXE: mutually exclusive exons, RI: retained intron, and SE: skipped exon also called cassette exons).


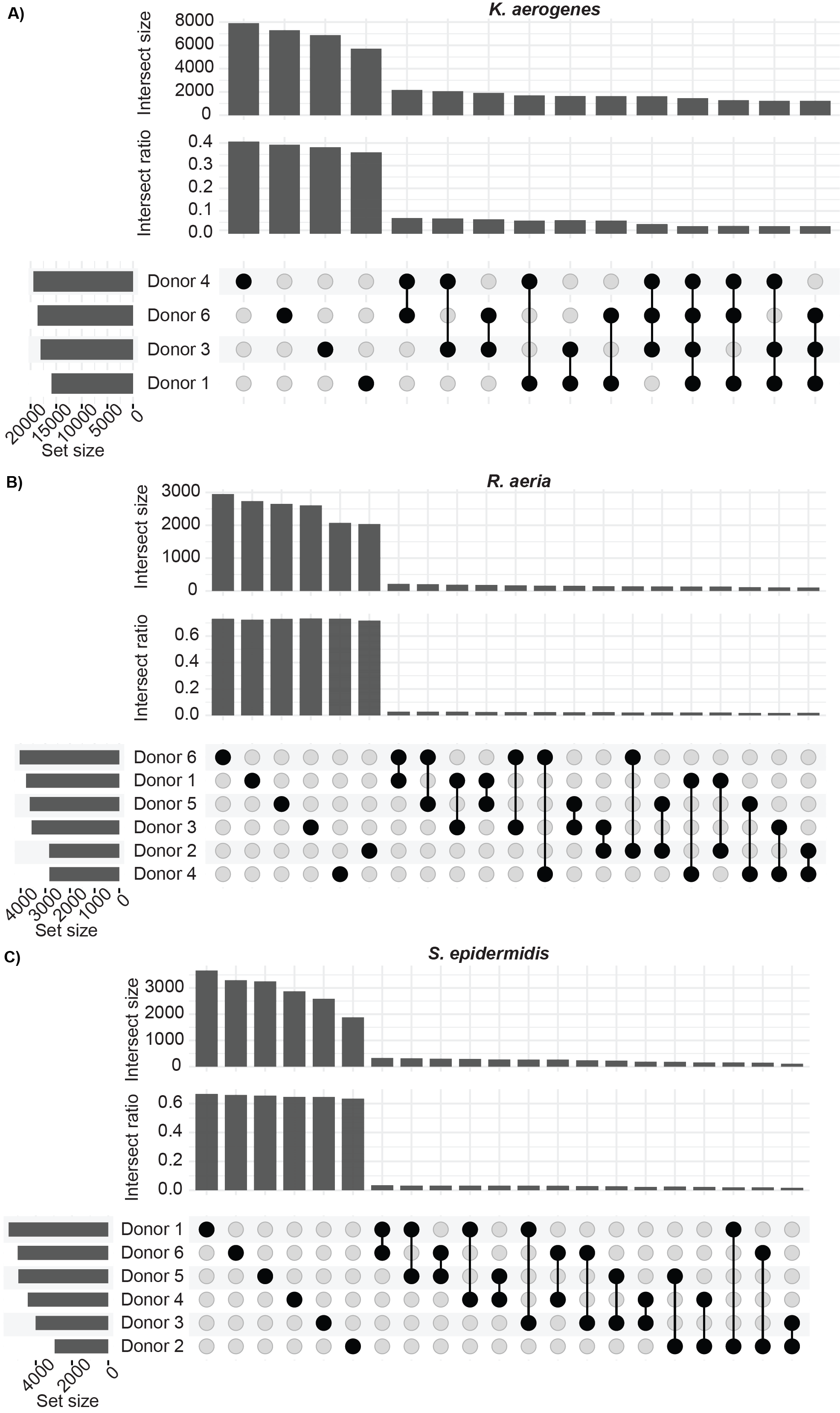


**Figure S10. Differentially splicing events shared between donors for each microbe.** Upset plot of splicing events depicting how many events are conserved between donors, shown for each microbe: *K. aerogenes* (**A**), *R. aeria* (**B**), and *S. epidermidis* (**C**). Set size represents the total number of splicing events. Donors are sorted by decreasing set size. Intersect size represents the number of splicing events shared between the indicated donors (black points connected by lines). Only intersections with a size of at least 50 are displayed. Intersect ratio normalizes the intersect size by union size (all unique elements across the sets).

**Supplementary Tables**

**Table S1**: List of donors who provided epithelial cells. For each sample, provided is their age, sex, and race.

**Table S2**: Transepithelial electrical resistance (TEER) measurements collected during ALI maturation for each donor.

**Table S3**: CFUs (colony forming units, a measure of live bacteria) from the inoculum and washed off of the ALI at each harvest for each microbial treatment/vehicle control.

**Table S4**: Number of differentially expressed genes (DEGs) that were upregulated and downregulated in response to each microbial treatment.

**Table S5a**: Reactome pathway analysis of genes differentially expressed between donors in response to microbial treatment following 12 hours of colonization.

**Table S5b**: Reactome pathway analysis of genes differentially expressed between donors in response to microbial treatment following 24 hours of colonization.

**Table S5c**: Reactome pathway analysis of genes differentially expressed between donors in response to microbial treatment following 48 hours of colonization.

**Table S6**: The gene lists used.

**Table S7**: The gene scores calculated for each sample for each gene list.

**Table S8**: Number of differential splicing events (FDR < 0.05 and absolute PSI > 0.1) for each microbial treatment. A3SS = alternative 3’ splice site, A5SS = alternative 5’ splice site, MXE = mutually exclusive exons, RI = retained intron, SE = skipped/cassette exon.

**Table S9**: Number of differentially spliced genes, differentially expressed genes (identified from the deeper read depth), and genes that were differentially spliced and differentially expressed.

**Table S10**: Reactome pathway analysis of differentially spliced genes.

**Table S11**: Number of functional domain changes for each microbial treatment. GoD = gain of domain, LoD = loss of domain, NMD = nonsense-mediated decay, other_region = other functional change, no_change = no functional change.
